# Data quality in Brazilian population-based cancer registries for gastrointestinal cancers

**DOI:** 10.1186/s12885-024-12477-2

**Published:** 2024-07-19

**Authors:** Diego Rodrigues Mendonça e Silva, Max Moura de Oliveira, Maria Paula Curado

**Affiliations:** 1 Postgraduate Program in Epidemiology, School of Public Health, University of São Paulo, São Paulo, SP Brazil; 2 Hospital Cancer Registry, A.C. Camargo Cancer Center, São Paulo, SP Brazil; 3 Department of Collective Health, Institute of Tropical Pathology and Public Health, Federal University of Goias, Goiânia, GO Brazil; 4 Group of Epidemiology and Statistics on Cancer, A.C. Camargo Cancer Center, São Paulo, SP Brazil

**Keywords:** Incidence, Data quality, Monitoring, Epidemiological surveillance

## Abstract

**Background:**

Population-based cancer registries (PBCRs) are the primary source of information for cancer surveillance and monitoring. Currently, there are 30 active PBCRs in Brazil. The objective of this study was to analyze the data quality of five gastrointestinal cancers (esophagus, stomach, colorectal, liver, and pancreas) according to the criteria of comparability, validity, completeness, and timeliness in Brazilian cancer registries.

**Methods:**

This study included data from Brazilian PBCRs with more than ten years of historical data starting in the year 2000, regardless of the type of defined geographical coverage (state, metropolitan region, or capital), totaling 16 registries. Brazilian PBCRs were evaluated based on four international data quality criteria: comparability, validity (accuracy), completeness, and timeliness. All cancer cases were analyzed, except for nonmelanoma skin cancer cases (C44) and five gastrointestinal tumors (esophageal cancer, stomach cancer, colorectal cancer, liver cancer, and pancreatic cancer) per cancer registry and sex, according to the available period.

**Results:**

The 16 Brazilian PBCRs represent 17% of the population (36 million inhabitants in 2021) according to data from 2000 to 2018. There was a variation in the incidence in the historical series ranging from 12 to 19 years. The proportion of morphologically verified (MV%) cases varied from 74.3% (Manaus) to 94.8% (Aracaju), and the proportion of incidentally reported death certificate only (DCO%) cases varied from 3.0% (São Paulo) to 23.9% (Espírito Santo). High-lethality malignant neoplasms, such as liver and pancreas, had DCO percentages greater than 30% in most cancer registries. The sixteen registries have more than a 48-month delay in data release compared to the 2022 calendar year.

**Conclusion:**

The studied Brazilian cancer registries met international comparability criteria; however, half of the registries showed indices below the expected levels for validity and completeness criteria for high-lethality tumors such as liver and pancreas tumors, in addition to a long delay in data availability and disclosure. Significant efforts are necessary to ensure the operational and stability of the PBCR in Brazil, which continues to be a tool for monitoring cancer incidence and assessing national cancer control policies.

**Supplementary Information:**

The online version contains supplementary material available at 10.1186/s12885-024-12477-2.

## Background

Population-based cancer registries (PBCRs) are crucial sources of information for cancer incidence, trend monitoring, and contributions to epidemiological studies, analytical research, and cancer estimates [1]. They operate in a country or region, utilizing data from all diagnosed cancer patients within a defined population and time interval. The proportion of the world’s population covered by registries included in Volume XI of CI5 (Cancer Incidence in Five Continents), published by the International Agency for Research on Cancer (IARC), was 15%, with coverage of 1% in Africa, 8% in Central and South America, 98% in North America, 7% in Asia, 46% in Europe, and 77% in Oceania [2].

Governmental and nongovernmental cancer institutions, along with research groups, use incidence data to generate cancer incidence estimates. Different methodologies can be employed for estimating incidence, and the validity of these estimates depends on the degree of representativeness and quality of the source information. Countries with lower and middle-income levels often exhibit lower overall quality and coverage of cancer registries than do higher-income countries [3, 4]. Globocan (IARC) emphasizes short-term forecasting and the use of modelled mortality-to-incidence (M: I) or incidence-to-mortality (I: M) ratios when applicable for national projections [5, 6]. The Global Burden of Disease (GBD) study generates incidence estimates by dividing mortality estimates (after correction) by mortality-to-incidence ratio (MIR) estimates for national and regional projections [6]. In Brazil, theNational Cancer Institute of Brazil (INCA) employs a methodology similar to that of Globocan (IARC) [5], using a short-term model (predicting up to five years) based on linear time prediction models. This approach is applied to capitals and states with incidence information covering a minimum of six years and up to a maximum of 15 years of historical data, with at least 50 cancer cases per year (across all age groups). For areas without PBCR, incidence is estimated using the median I/M ratio for the geographical region where estimation is desired [7].

Brazil, a continental country with 27 federative units and over 213 million inhabitants [8], faces the challenge of maintaining continuous operational and data quality in the PBCR. Currently, there are 30 PBCRs with population coverage at the state, capital, metropolitan region, health region, or municipal level [7]. Ten Brazilian PBCRs participated by including their data in the IARC publication (CI5), the gold standard for registry quality, from the first to the tenth volume [9]. The latest volume XI included six Brazilian PBCRs [2].

Given the importance of using PBCR data for cancer burden monitoring through incidence, its use in incidence estimates and the absence of studies describing data quality, this study aimed to analyze the quality of data from Brazilian cancer registry considering the gastrointestinal cancers, as this group are major contributors of the top seven causes of cancer-related death, including colorectal, stomach liver, pancreatic and esophageal cancer. Overall, common gastrointestinal tract cancers accounted for 35% of neoplasms-related [10]. We analyzed the comparability, validity, completeness, and timeliness criteria foresophageal, stomach, colorectal, liver, and pancreatic cancers.

## Methods

This is a study based on data from population-based cancer registries (PBCRs) obtained through the National Cancer Institute on Jan 8, 2023, from the link https://www.gov.br/inca/pt-br/assuntos/cancer/numeros/registros/base-populacional, with the database’s latest update on Nov 25, 2022. The INCA database contains information from 33 cancer registries, of which 30 PBCRs are currently active [7]. The inclusion criteria for this study were PBCRs with more than ten years of information, starting in the year 2000, and population coverage at the state, metropolitan region, or capital level (Supplementary Table 1). Brazilian PBCRs were evaluated against four internationally defined quality criteria: comparability, validity (accuracy), completeness, and timeliness [11, 12]. The analysis criteria were as follows:

### Comparability

The comparability of statistics generated over time requires standardizing practices related to the classification and coding of new cases and ensuring consistency in incidence definitions. The standard for cancer classification and coding is the International Classification of Diseases for Oncology, published by the World Health Organization (WHO), which provides standards for topography, morphology, and tumor behavior coding [11, 12]. Comparability was verified based on the guidelines to which Brazilian registries adhere.

### Validity

Data validity (accuracy) can be improved by consistency checks performed through data entry methods [12], as described in this study through diagnostic method criteria (morphologically verified and death certificate only) and missing information analysis. Validity was analyzed by cancer registry, sex, and overall, for all incident cases except nonmelanoma skin cancer (NMSC, C44) and for cancers of the esophagus (C15), stomach (C16), colorectum (C18-C20), liver (C22), and pancreas (C25), classified according to the International Classification of Diseases for Oncology, 3rd edition (ICD-O3). The inclusion criteria were as follows:

### Proportion of morphologically verified (MV%)

For most cases, the gold standard for diagnosis is a histological report released by a pathologist. Cancer registries may also use other diagnostic sources, such as hematologic tests, imaging, or clinical information. Therefore, the validity index is often the percentage of morphologically verified cases [12]. Morphologically verified cases were identified by examining the proportion of MV compared to other notification types (clinical, imaging, or death certificate) for incident cases. The recommendation is an MV% above 70% [2].

### Proportion of deaths certificate only (DCO%)

The proportion of cancers for which no information other than a death certificate mentioned cancer was available was another validity measure. Death certificate-only cases represent the residue of cases after all case identification strategies have been completed, for which no information other than a death certificate mentioning cancer could be obtained. Thus, a high percentage of DCO may indicate inappropriate case detection [12]. The DCO proportion was calculated in relation to other notification types, and the recommended DCO% was less than 20% [2].

### Proportion of patients of unknown age

Another variable commonly assessed for the proportion of missing values is age [11]. The greater the proportion of patients of unknown age, the greater the impact on incidence rate calculations since these patients may be excluded from incidence analyses. It is recommended that the absence of age information be less than 10% in the database [2].

### Proportion of unknown primary sites (C80)

Incidence rates for cancer in specific locations may be underestimated if a significant proportion of cases are recorded in the “unknown primary site” category. The proportion of cases attributed to category C80 can indicate the accuracy of obtaining the primary tumor diagnosis [11]. The proportion of cases coded as C80 was calculated to identify cases where the primary site of the tumor was unknown in relation to the total. It is recommended that the percentage of C80 be below 10% in the database [2].

### Completeness

Completeness can be analyzed using qualitative (or semiquantitative) methods that indicate the degree of completeness compared to other registries or over time or quantitative methods that provide a numerical assessment of how many eligible cases have been registered. In this study, completeness was analyzed through semiquantitative methods, such as the possibility that a relatively high MV% (close to 100%) may represent data collection incompleteness and failure to identify information sources. This is because the entry of a case can be identified from different sources [11], the stability of incidence over time, and the mortality-to-incidence ratio.

### Stability of incidence over time

Changes in the stability of incidence over time may indicate potential failures in the coverage of case notification sources in the absence of significant changes in the population or diagnostic and treatment practices [11]. For this analysis, standardized incidence rates were calculated for all cancers, except nonmelanoma skin cancer, by sex and PBCR according to the available data. Standardized rates were calculated using the direct method, with the Segi (1960) world population standard modified from Doll (1966). Population data were obtained from the Brazilian Institute of Geography and Statistics (IBGE), available on DATASUS, and extracted by geographic coverage of each PBCR by year, age group (5–5 years), and sex [8].

### Mortality-to-incidence ratio (M: I)

The mortality-to-incidence ratio is an important indicator used to report possible incompleteness. It compares the number of deaths, obtained from an external source to the registry (usually vital statistics systems), and the number of new cases of a specific cancer in the same time period [11]. The M: I ratio was calculated for each topography (esophagus, stomach, colorectum, liver, and pancreas) between the number of deaths and estimated new cases, based on the cumulative standardized rates in the incidence and mortality period, by sex and cancer registry. If the declaration of causes of death was accurate and incidence and survival were constant, the M: I ratio was equal to 1 minus the probability of survival. A ratio greater than 1 indicates underreporting [13]. The M: I ratio was calculated by the unbiased median estimate (mid-p) and unconditional maximum likelihood estimate (Wald).

### Timeliness

The prompt notification of cancer case information should be another priority for cancer registries. Timeliness is related to how quickly a registry can collect, process, and report sufficiently reliable and complete cancer data [12]. Timeliness was analyzed by calculating the time (in years) between the last year of information available for each registry and the current calendar year (2022). Timeliness depends on infrastructure, resources, and the work team, reflecting each cancer registry’s capacity to collect, process, and provide complete and accurate data.

### Software’s

The map was plotted in QGIS Development Team 2022, version 3.24.2-Tisler from April 15, 2022, a software with a General Public License [14].

The descriptive analyses and the incidence rate were performed in Microsoft Excel.

The Mortality-to-incidence ratio was performed in RStudio software version 1.2.5042, the “epitools” package, and the rate ratio (ratioratio) [13], the confidence intervals (95%) were calculated using exact methods (mid-p) and the normal approximation (Wald).

## Results

**Fig. 1:**
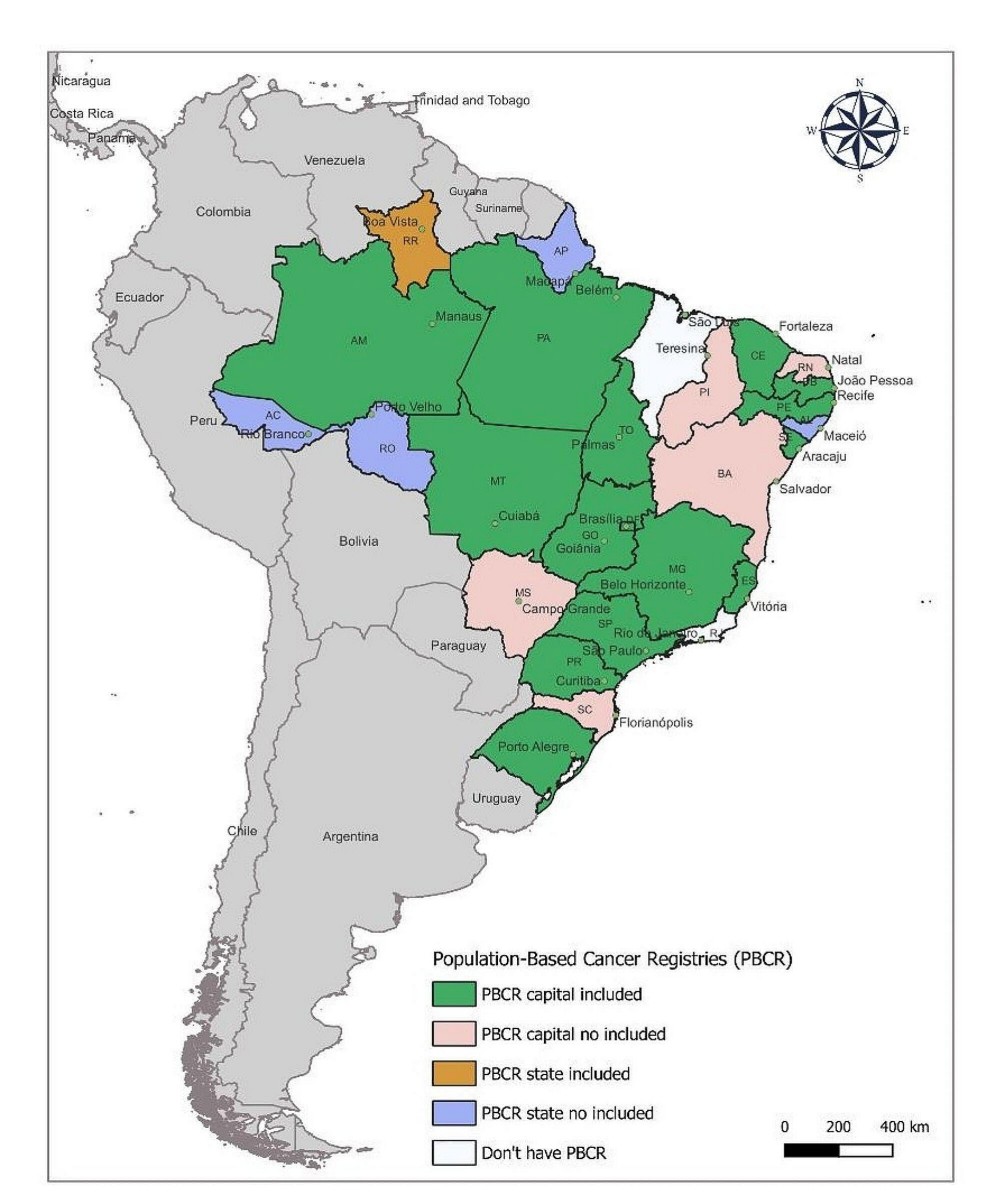
Geographic distribution of Population-Based Cancer Registries with statewide or capital coverage, included and not included in the study

Of the 27 Federative Units in Brazil, this study included PBCRs from 15 states and the Federal District; in nine states, the PBCRs were not included because they did not meet the eligibility criteria, and two states had no active registry. The 16 studied PBCRs, according to the data availability period, were as follows: Espírito Santo (2000–2012); Manaus (2000–2014); Roraima (2003–2014); Fortaleza and São Paulo (2000–2015); Aracaju (2000–2016); Belém, Curitiba, Federal District, Goiânia, João Pessoa, Palmas, Porto Alegre, and Recife (2000–2017); Belo Horizonte and Cuiabá (2000–2018) (Supplementary Table 1). The 33 cancer registries of the Brazilian population covered 26% of the population in 2021, approximately 56 million inhabitants. The 16 PBCRs included in this study (Fig. 1) represent 17% of the Brazilian population (36 million), with incident data available from 2000 to 2018 and a historical series ranging from 12 to 19 years, totaling approximately 1,300,000 cases, excluding nonmelanoma skin cancer, and over 300,000 cases of nonmelanoma skin cancer (Table 1).

The PBCR with the highest population coverage is São Paulo, representing 5.8% of the Brazilian population (Supplementary Table 1), with 531,943 incident cases between 2000 and 2015 (Table 1). The percentage of nonmelanoma skin cancer (NMSC) among the studied PBCRs varied from 6.9% in Espírito Santo to 35.9% in Goiânia. NMSC patients were excluded from the data quality analyses according to predefined criteria (Table 1).

### Comparability

Comparability is ensured by national and international norms and recommendations. All the Brazilian PBCRs follow the rules and recommendations from the National Cancer Institute of Brazil (INCA), that supports and assists Brazilian cancer registries through activities focused on registrar training, specific educational materials, operational support for computerized systems, and data publication. The INCA also standardizes practices disseminated through the Manual of Routines and Procedures for Population-Based Cancer Registries.

### Validity

**Fig. 2:**
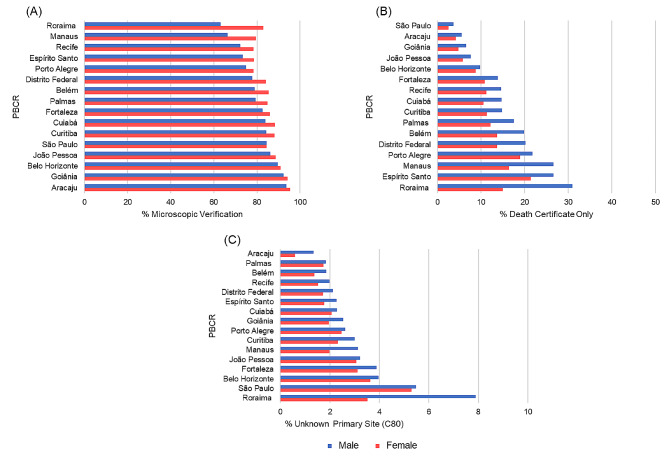
Proportion of **(A)** morphologically verified (MV%), **(B)** proportion of death certificate only (DCO%), and **(C)** proportion of primary site unknown (C80%), for all cancers except non-melanoma skin cancer, by Population-Based Cancer Registry and sex

The proportion of morphologically verified (MV%) varied from 74.3% in Manaus to 94.8% in Aracaju. The proportion of deaths certificate only (DCO%) varied from 3.0% in São Paulo to 23.9% in Espírito Santo. The percentage of unknown primary sites (C80) was greater in São Paulo (5.4%) and Roraima (5.2%) (Table 1). The indicators of morphology verification and death certificate-only status depended on the sex, with men having a greater proportion of cases identified by a death certificate above 20% in Porto Alegre, Manaus, Espírito Santo, and Roraima (Fig. 2).

The proportion of patients of unknown age was as recommended in all PBCRs, except for the PBCR of Roraima with 20.0% unknown age for all cancers except NMSC (Table 1).

All Brazilian PBCRs showed the quality in the indicator of the proportion of unknown primary sites (C80) as recommended to be below 10% (Table 1). The highest proportion of unknown primary sites (C80) was also observed in males in all registries (Fig. 2).

**Table 1:** Distribution of incident cases regarding the total and proportion of non-melanoma skin cancer (NMSC) cases, proportion of cases registered by morphologically verified (MV%), percentage of cases by death certificate only (DCO%), age ignored and unknown primary site (C80), except NMSC, by Brazilian PBCRs, 2000–2018

		All cases (C00-C80)			Cases, except non-melanoma skin cancer (C00-C80, except C44)						
Population-Based Cancer Registry (PBCR)	Period of diagnosis	Total	NMSC	NMSC/ total	All cases, except NMSC	MV%	DCO%	Age ignored		Unknown primary site (C80)	
	**Years**	**N**	**N**	**%**	**N**			**N**	**%**	**N**	**%**
**North**											
Belém	2000–2017	59,807	6,396	10.7	53,411	82.8	16.3	1,285	2.4	841	1.6
Manaus	2000–2014	38,135	3,439	9.0	34,696	74.3	20.5	391	1.1	847	2.4
Palmas	2000–2017	4,929	656	13.3	4,272	82.3	14.6	47	1.1	76	1.8
Roraima	2003–2014	5,745	1,045	18.2	4,700	75.1	21.3	941	20.0	246	5.2
**Northeast**											
Aracaju	2000–2016	43,376	14,685	33.9	28,691	94.8	4.6	40	0.1	240	0.8
João Pessoa	2000–2017	27,578	3,458	12.5	24,118	87.7	6.6	20	0.1	754	3.1
Fortaleza	2000–2015	93,694	18,748	20.0	74,940	84.6	12.1	1,214	1.6	2572	3.4
Recife	2000–2017	67,708	8,182	12.1	59,526	76.0	12.5	116	0.2	1,015	1.7
**Central-West**											
Cuiabá	2000–2018	35,524	7,956	22.4	27,566	86.2	12.5	192	0.7	597	2.2
Distrito Federal	2000–2017	95,658	14,227	14.9	81.422	81.5	16.3	7,378	9.1	1,533	1.9
Goiânia	2000–2017	87,693	31,449	35.9	56,244	93.2	5.5	42	0.1	1,254	2.2
**Southeast**											
Belo Horizonte	2000–2018	173,753	37,374	21.5	136,372	90.3	92.2	649	0.5	5,157	3.8
Espírito Santo	2000–2012	27,249	1,877	6.9	25,371	76.0	23.9	46	0.2	510	2.0
São Paulo	2000–2015	656,917	124,974	19.0	531,943	84.4	3.0	35,304	6.6	28,620	5.4
**South**											
Curitiba	2000–2017	92,399	15,449	16.7	76,950	86.4	12.8	29	0.0	2,018	2.6
Porto Alegre	2000–2017	105,817	21,183	20.0	84,591	76.9	20.2	2,226	2.6	2,143	2.5

NMSC: nonmelanoma skin cancer; MV%: proportion of morphologically verified; DCO%: proportion of death certificate only

C00-C80: Topography in International Classification of Disease in Oncology 3rd edition

PBCRs: Population-Based Cancer Registries

**Fig. 3:**
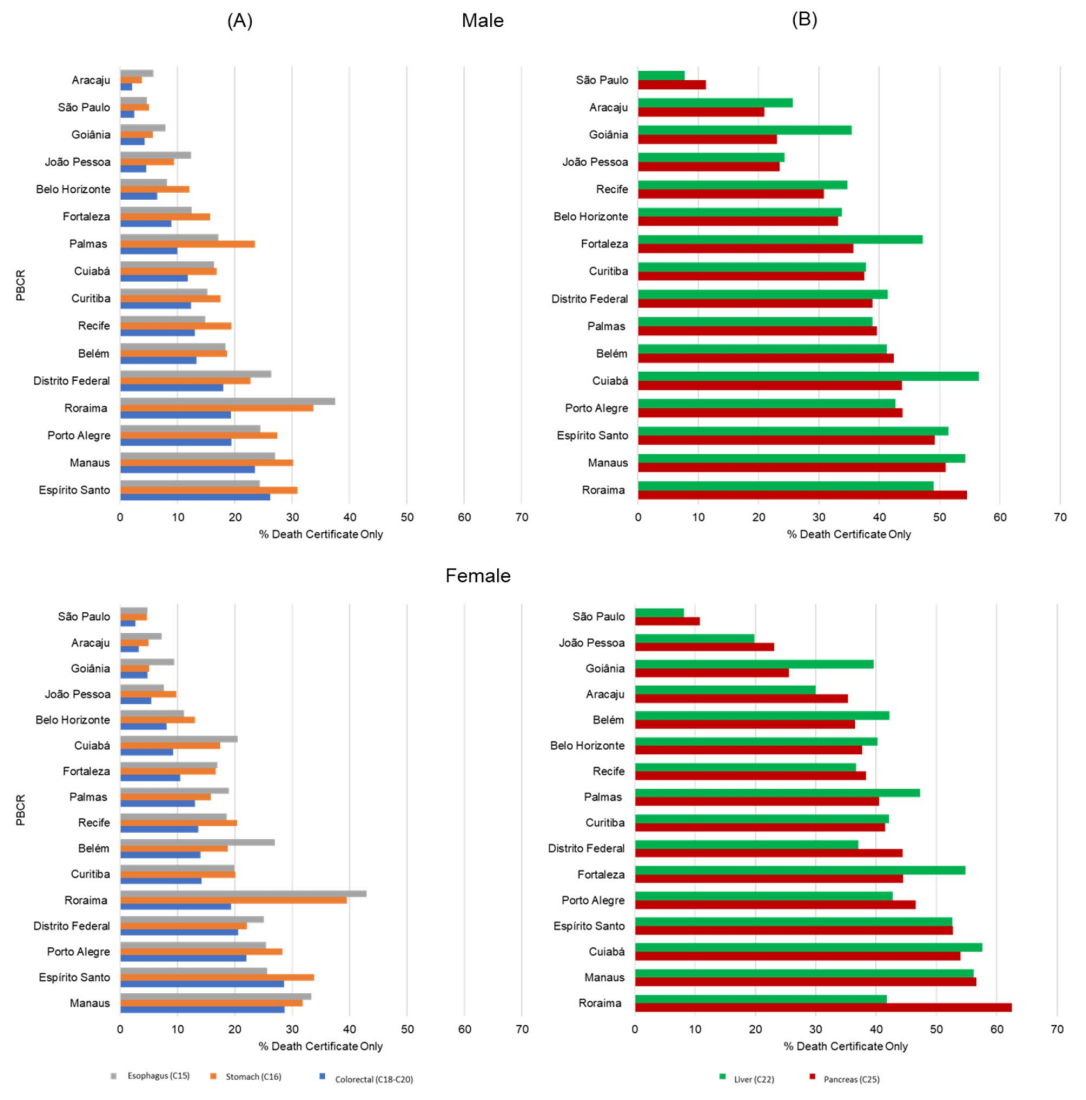
Proportion of death certificate only (DCO%) for tumors of the gastrointestinal tract **(A)** esophagus, stomach, colorectal, and **(B)** accessory organs liver and pancreas, by Population-Based Cancer Registry and sex

For esophageal, stomach, and colorectal cancers, higher morphologically verified and lower death certificate-only values were observed. However, for liver and pancreas cancers, morphologically verified values were below 80%, and death certificate-only values were above 30% in most registries (Table 2). In Roraima, the highest percentages of death certificate-only for esophagus and stomach cancers in both sexes and pancreas cancer for females were observed, while for liver cancer, the highest percentage of death certificate-only was observed in Cuiabá in males (Fig. 3).

### Completeness

The highest proportion of MV was observed in PBCR of Aracaju with 94.8%, followed by Goiania (93.2%). These which higher MV% close to 100%, may represent data collection incompleteness and failure to identify information sources.

**Fig. 4:**
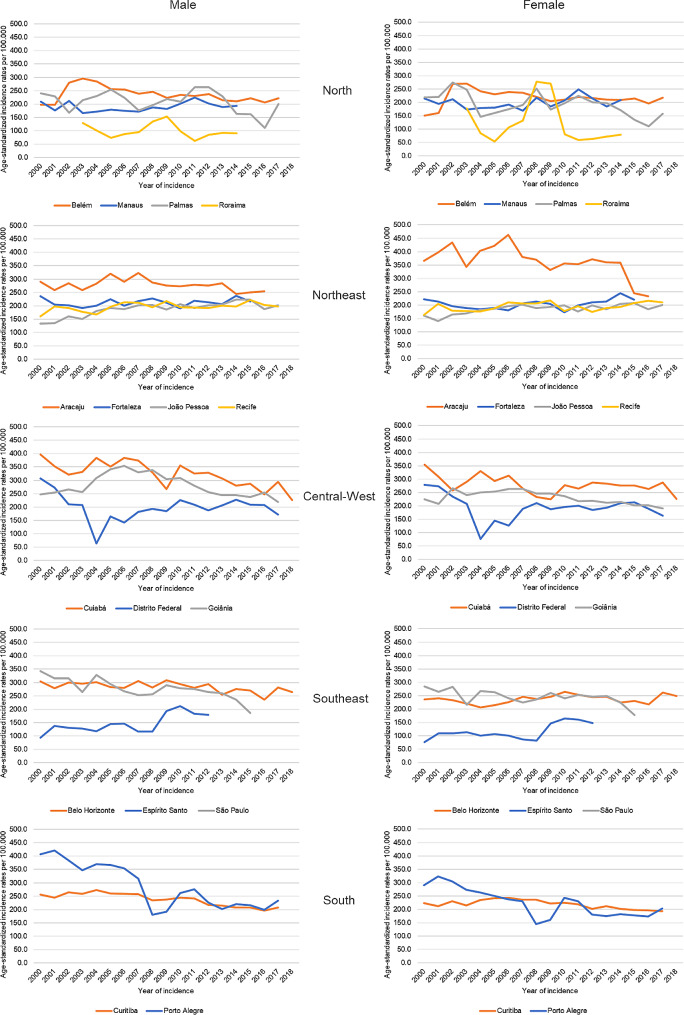
Age-standardized incidence rates for all cancers, excluding non-melanoma skin cancer, by Population-Based Cancer Registry and sex, from 2000 to 2018

In the stability of incidence over time, the ten-year or greater incidence rates for all cancer cases, except nonmelanoma skin cancer, were stable in the PBCRs of Fortaleza, João Pessoa, Recife, and Belo Horizonte among men and women. According to Fig. 4, it showed that twelve Brazilian PBCRs do not have stability of incidence over time.

**Fig. 5:**
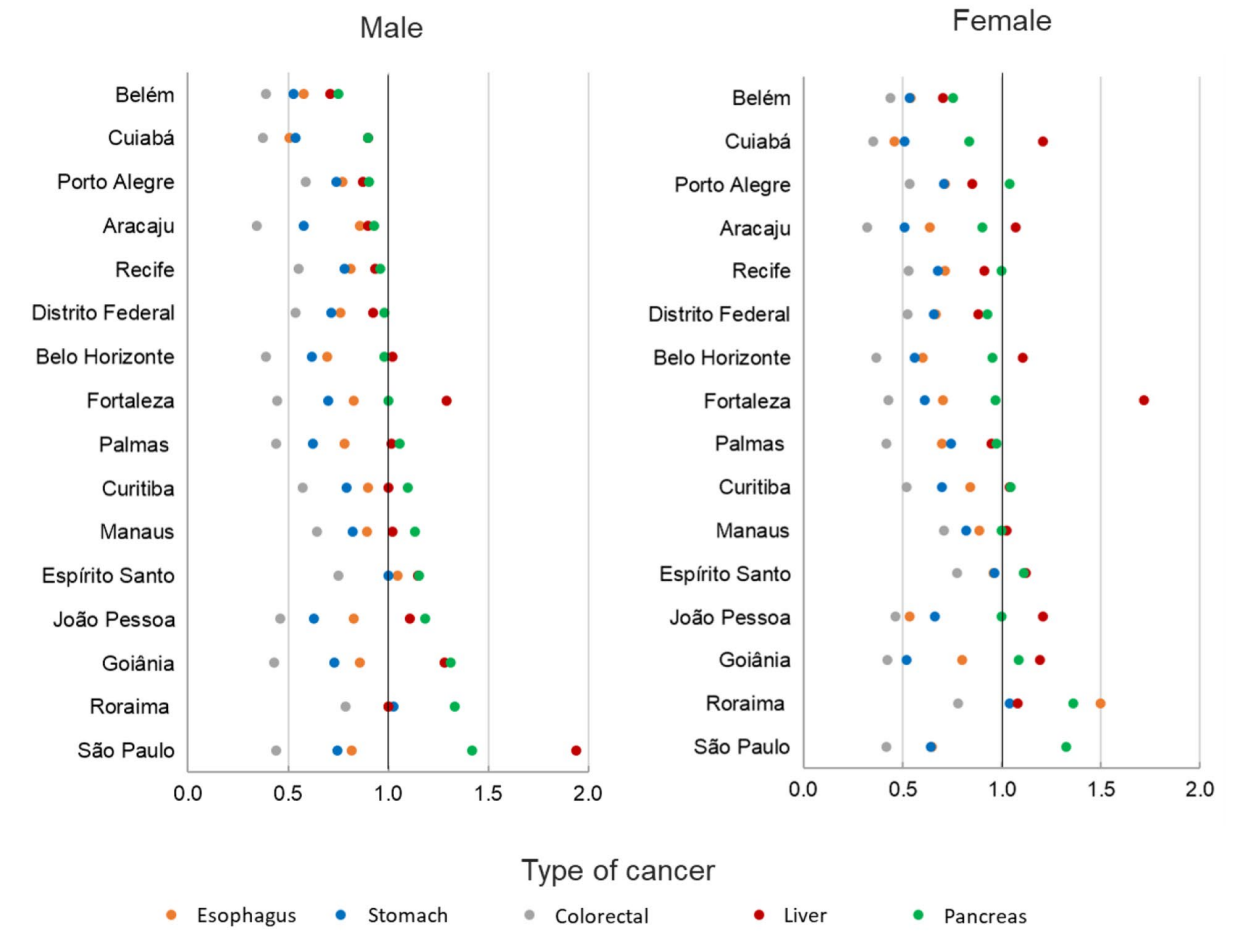
Comparison of the mortality-to-incidence ratio for esophageal, stomach, colorectal, liver, and pancreatic cancers by sex and Population-Based Cancer Registry

For the mortality-to-incidence ratio (M: I) we observed M: I exceeding 1 for liver cancer in Espírito Santo (M: I 1.1; 95% CI 1.0;1.3), Fortaleza, Goiânia, and São Paulo (M: I 1.9; 95% CI 1.9;2.0) in males. While, between females, M:I was larger than 1 in Belo Horizonte, Espírito Santo, Cuiabá, Fortaleza, Goiânia, João Pessoa, and São Paulo. For pancreatic cancer, an M: I greater than 1 was observed in both sexes in Curitiba, Espírito Santo, Goiânia, and São Paulo, including João Pessoa and Manaus among males and Roraima among females  (Fig. 5).

**Table 2:** Distribution of incident cases by the proportion of cases registered by morphologically verified (MV%), percentage of cases by death certificate only (DCO%), according to cancer type and Brazilian PBCRs, from 2000 to 2018

	Esophagus (C15)				Stomach (C16)				Colorectal (C18-C20)				Liver (C22)				Pancreas (C25)			
PBCR	Cases	% all cases	MV%	DCO	Cases	% all cases	MV%	DCO%	Cases	% all cases	MV%	DCO%	Cases	% all cases	MVMV%	%DCO	Cases	% all cases	MV%	DCO%
**North**																				
Belém	662	1.2	78.5	20.7	5,593	10.5	80.5	18.7	3,010	5.6	85.5	13.7	1,376	2.6	55.5	41.6	1,059	2.0	56.5	39.5
Manaus	410	1.2	59.5	28.3	2,811	8.1	60.5	30.7	1,311	3.8	66.7	26.2	1,008	2.9	34.4	55.1	614	1.8	32.1	53.9
Palmas	62	1.5	77.4	17.7	189	4.4	77.2	21.2	320	7.5	85.6	11.6	109	2.6	53.2	43.1	95	2.2	57.9	40.0
Roraima	55	1.2	61.8	38.2	265	5.6	57.0	35.5	145	3.1	77.9	19.3	171	3.6	46.2	46.2	65	1.4	35.4	58.5
**Northeast**																				
Aracaju	209	0.7	92.3	6.2	719	2.5	94.4	4.3	1,265	4.4	97.0	2.8	374	1.3	68.7	27.5	361	1.3	66.8	29.6
Fortaleza	1,273	1.7	84.4	13.7	4,225	5.6	81.7	16.1	4,566	6.1	88.4	9.8	746	1.0	48.0	50.3	1,315	1.8	51.6	40.6
João Pessoa	360	1.5	81.9	10.8	1,111	4.6	82.3	9.5	1,331	5.5	88.7	5.0	441	1.8	56.7	22.2	443	1.8	56.2	23.3
Recife	927	1.6	70.6	16.0	2,340	3.9	65.3	19.8	3,532	5.9	70.6	13.3	1,806	3.0	46.0	35.7	1,423	2.4	43.2	35.1
**Central-West**																				
Cuiabá	568	2.1	81.2	17.3	1,251	4.5	81.9	17.0	1,955	7.1	87.7	10.5	407	1.5	41.5	56.8	430	1.6	47.2	48.8
Distrito Federal	1,623	2.0	72.3	26.1	3,921	4.8	75.4	22.4	6,261	7.7	79.5	19.4	1,616	2.0	59.1	39.5	1,633	2.0	56.4	41.8
Goiânia	787	1.4	90.9	8.3	2,306	4.1	93.3	5.5	4,705	8.4	94.8	4.5	732	1.3	56.7	36.9	880	1.6	70.8	24.3
**Southeast**																				
Belo Horizonte	3,039	2.2	90.0	9.0	5,898	4.3	86.7	12.5	11,523	8.4	92.2	7.4	1,755	1.3	63.1	36.1	2,653	1.9	62.8	35.6
Espírito Santo	1,112	4.4	75.4	24.6	1,653	6.5	68.1	31.9	1,825	7.2	72.4	27.5	663	2.6	48.1	51.9	615	2.4	49.1	50.9
São Paulo	9,110	1.7	85.5	4.7	24,294	4.6	84.4	4.9	53,170	10.0	85.8	2.6	3,975	0.7	89.7	7.9	7,892	1.5	59.2	11.0
**South**																				
Curitiba	1,684	2.2	81.9	16.2	3,717	4.8	80.6	18.5	7,100	9.2	85.7	13.3	1,385	1.8	60.1	39.4	1,862	2.4	59.0	39.6
Porto Alegre	2,261	2.7	71.5	24.7	3,037	3.6	69.0	27.8	7,900	9.3	76.6	20.8	2,417	2.9	52.8	42.7	2,298	2.7	49.1	45.4

PBCRs: Population-Based Cancer Registries; MV%: proportion of morphologically verified; DCO%: proportion of death certificate only

### Timeliness

According to the timeliness criterion, all PBCRs presented delays exceeding 48 months in making incidence data available compared to the calendar year 2022, with the shortest delay being 4 years for Cuiabá and Belo Horizonte and the longest delay being 10 years for Espírito Santo (Table 1).

## Discussion

This study assessed four data quality criteria — comparability, validity, completeness, and timeliness — in 16 Brazilian Population-Based Cancer Registries (PBCRs) for all cancers except nonmelanoma skin cancer and for esophageal, stomach, colorectal, liver, and pancreatic cancers. Comparability is ensured by national and international norms and recommendations [15]. The proportions of morphologically verified (MV%) and death certificate-only (DCO%) patients were adequately stable for all cancers except nonmelanoma skin cancer. However, the completeness criteria were heterogeneous, as indicated by the stability of incidence over time, with only four PBCRs demonstrating stable rates. Validity (MV% and DCO%) and completeness (mortality-to-incidence ratio) by cancer type showed a low percentage of MV and high DCO% for lethal cancers such as liver and pancreas cancers. Seven out of the sixteen studied registries had an M: I ratio greater than 1. According to the timeliness criterion, all PBCRs presented delays exceeding 48 months in incidence data available.

The population coverage of the Brazilian PBCR, at 17% of the national population in this study, contrasts with the 40% coverage observed in China and the 96% coverage in the United States, with nearly 100% in the United Kingdom, Australia, and South Korea [16]. Our findings reflect the lack of investments in cancer registries in Brazil, while China increased its cancer registries from 43 in 2008 to 574 in 2019, aligning with increased funding for expansion [17]. In Brazil, national resources supporting the PBCR come from the Ministry of Health’s Ordinance 2.607/GM, dated December 28, 2005, with current values equivalent to just over R$1 million annually (less than US$200,000 annually) [18]. These values, set according to the 2005 population size among 22 federative units of Brazil, are insufficient for the continuity and expansion of cancer registries. For São Paulo, the largest Brazilian city with more than 12 million inhabitants, the fixed amount was R$10,000.00 monthly (equivalent to less than US$2,000.00) in 2005, corrected to R$15,000.00 (less than US$3,000.00) monthly in 2014 [19]. This is insufficient for PBCR funding, and as a result, São Paulo relied on financial support from the Municipal Health Secretariat and infrastructure support from the University of São Paulo’s School of Public Health [20]. However, the financial support agreement with the municipal secretariat was discontinued in 2019. Other Brazilian PBCRs, such as the one in Goiânia, exist and sustain their activities through agreements and resources with other institutions [21].

The National Cancer Institute of Brazil (INCA) supports and assists Brazilian cancer registries through activities focused on registrar training, specific educational materials, operational support for computerized systems, and data publication. The INCA also standardizes practices disseminated through the Manual of Routines and Procedures for Population-Based Cancer Registries [15], based on principles and methods developed by the International Agency for Research on Cancer (IARC) and the International Association of Cancer Registries (IACR), ensuring international comparability of incidence data [7]. However, variations in the inclusion criteria for nonmelanoma skin cancers (NMSCs) were observed among the studied registries, possibly leading to underreporting or overreporting of these tumors.

The percentage of MV, a validity criterion, in all analyzed cancer registries is comparable to that of the European and American PBCRs, which have high data validity [22, 23] and follow IARC recommendations of above 70% for all cancers except NMSC [2]. These results showed Brazilian PBCRs have access to the anatomical pathology laboratory. However, when analyzing MV% by cancer type, Manaus’s PBCR showed a low percentage of MV for esophageal, stomach, colorectal, and pancreatic tumors, likely due to health service supply and coverage issues and a lack of integration among services. Roraima’s statewide PBCR also demonstrates difficulty in identifying cases at the primary diagnostic source [24]. For pancreatic cancer, the percentage of MV was less than 71% in all sixteen Brazilian registries, similar to liver cancer, except in São Paulo (89.7%). This might indicate incomplete registration of clinical cases or inadequate histological verification. Indeed, a low MV percentage for pancreatic cancer (63%) was also observed in Finland [25], which was lower than that in Norway [22] and Iceland [26] due the high-lethality of this cancer, this may be reason why, which MV is low.

The percentage of DCO in some registries in the Americas is less than 5%, as recommended locally. Percentages below 5% for DCO cases were observed in the PBCRs of São Paulo and Aracaju when analyzing the total cases. However, Espírito Santo (metropolitan), Roraima (statewide), Manaus (capital), and Porto Alegre (capital) PBCRs had DCO above the recommended < 20% for all neoplasms except nonmelanoma skin cancer [2]. A high DCO% for liver and pancreatic tumors may indicate difficulty in identifying these neoplasms at primary sources or the absence of clinical or pathological diagnoses. For tumors such as esophageal, stomach, and colorectal cancer, there was a greater MV% and lower DCO%, indicating greater access to diagnosis and more information, as the incidence of these neoplasms has increased in the population due to increased exposure to risk factors [2].

All sixteen cancer registries in this study had an unknown primary site percentage (C80) less than 6%, fulfilling the recommended < 10%. In Finland, 1.9% of cases were registered with an unknown primary location, and there was a strong association between C80% and age, possibly due to diagnostic [25]. The presence of an unknown primary site (C80) is associated with a late-stage cancer diagnosis, where the primary neoplasm is not identified, and a lack of information in medical records and pathological reports of the primary tumor [11].

Stability in incidence over time was observed for cancer registries in Fortaleza, João Pessoa, Recife, and Belo Horizonte. A Finnish study also described stability in rates without severe fluctuations [25], as observed in other Nordic countries [27]. However, most Brazilian registries showed significant fluctuations or declining trends in rates.

This may indicate potential failures in case notification source coverage [10], frequent change in the team, difficulty in accessing the sources, lack of investments on resources and infrastructure. There are 20% increase in incidence in the last decade, a continuous increase in the coming decades is expected [28], so stability or increase in incidence over time was expected.

The mortality-to-incidence ratio (M: I), a measure of completeness, calculated by obtaining mortality data from an independent source, usually Vital Statistics Systems [12], differs in Brazil, where it is provided by the Mortality Information System (SIM) [29], with national coverage and high-quality levels [30]. For pancreatic cancer, the expected M: I ratio is close to 1 due to the high-lethality of the tumor. A study on pancreatic cancer in Brazil and China revealed an inversion with higher mortality compared to incidence, possibly due to methods used by the Global Burden of Disease (GBD) 2019 study, leading to an excess of deaths and/or revealing the underestimation of incidence [32]. Thus, there may be underreporting of incident cases in Brazilian registries. An M: I greater than 1 for pancreatic cancer was observed in both sexes in this study in the PBCRs of Curitiba, Espírito Santo, Goiânia, and São Paulo, including João Pessoa and Manaus among men and Roraima among women. In all these locations, mortality rates were higher than incidence rates, indicating underreporting, as DCO cases represent the residue of cases after all case identification strategies have been completed, for which no other information beyond a death certificate mentioning cancer could be obtained [11].

Finally, the maximum lag time recommended for the evaluation of the consistency and disclosure of cancer incidence data in Brazil, conditioned on the right to receive the funding provided in ordinance No. 183 of January 30, 2014, is a maximum of two calendar years [18]. None of the studied cancer registries met this requirement. The delay in disclosing incidence data between 4 and 10 years compared to the calendar year (2022) limits the estimates produced from these data. Because of the insufficient resources and lack of investments from the government, the Brazilian PBCRs have fragility to continuing the routine activities and work fast due to lack of permanent policy. However, we believe that to reduce this timeliness, partnerships could be established between public and private institutions. Furthermore, technological resources could be incorporated in the routine registry, thus improving the PBCRs and reduce the delay of incidence data. In some European countries, electronic data capture has accelerated the registration process, with a median latency of 18 months for case verification and 3 to 6 months for data publication [25, 32]. Networks such as the Surveillance, Epidemiology, and End Results (SEER) and the North American Association of Central Cancer Registries reported data collected within 22–24 months after the end of the diagnosis [11].

Limitations of this study include the absence of incidence data in all Brazilian capitals, lack of homogeneity among available data periods, and significant delay compared to the calendar year. We do not analyze the 5th quality criterion on ‘quality indices for survival data’, since these results were not available in the dataset or accessible to us. The variable stage was not analyzed because it is not collected as standard in Brazilian PCBRs. As a strength, the analysis was conducted for 16 Brazilian registries with a broad population distribution, including over 1 million cancer cases (excluding nonmelanoma skin cancer) and over 18 years of incidence data.

## Conclusions

The quality of the Brazilian cancer registries met the comparability and validity criteria. However, when analyzing specific tumor groups with high-lethality, such as pancreatic and liver cancer, potential underreporting was observed, likely related to the natural history of these cancers. Factors such as access to sources such as hospitals and pathology laboratories, partnerships, financial support, and periodic registrar training can contribute to reducing delays in providing cancer incidence data in Brazil. Currently, there are disparities in the quality of available cancer data in Brazil, and greater efforts are needed to make incidence data available periodically and updated. A PBCR is an effective instrument for public health monitoring, whose costs are low compared to the benefits generated for the population. Cancer registry data, through trends in incidence, cancer survival, and mortality data, can be used to periodically assess overall progress in cancer control in the country. Therefore, ensuring the stability and sustainability of population-based cancer registries in Brazil will provide a policy that benefits the Brazilian population in cancer control.

### Electronic supplementary material

Below is the link to the electronic supplementary material.

Supplementary Material 1

## Data Availability

Public data available at https://www.gov.br/inca/pt-br/assuntos/cancer/numeros/registros/base-populacional.
