# Supplementary material for: Data quality in Brazilian population-based cancer registries for gastrointestinal cancers
Source: BMC Cancer. 2024 Jul 19;24:870. doi: 10.1186/s12885-024-12477-2 (PMC11264711; doi:10.1186/s12885-024-12477-2)
Supplement: Supplementary file 1 — Supplementary Material 1 [file 12885_2024_12477_MOESM1_ESM.docx]

**Supplementary Information**

| Supplementary Table 1. Description of the incidence database from Brazilian Population-Based Cancer Registries made available by the National Cancer Institute, according to region, period of available data, number of years registered, total cases, population and geographic coverage, and inclusion in the study. | | | | | | | | | |
| --- | --- | --- | --- | --- | --- | --- | --- | --- | --- |
| Region / PBCR | State | Years available in the registry* | Number of years recorded | All cases | Average/year | Population coverage (2021) | Percentage of the Brazilian population covered | Geographic coverage | Inclusion in the study** |
| **North** |  |  |  |  |  |  |  |  |  |
| Acre | AC | 2010-2017 | 8 | 6,031 | 754 | 906,876 | 0.4% | State | No |
| Amapá | AP | 2016 | 1 | 900 | 900 | 877,613 | 0.4% | State | No |
| Belém | PA | 2000-2017 | 18 | 59,807 | 3,323 | 1,506,420 | 0.7% | Capital | Yes |
| Manaus | AM | 2000-2014 | 15 | 38,135 | 2,542 | 2,255,903 | 1.1% | Capital | Yes |
| Palmas | TO | 2000-2017 | 18 | 4,929 | 274 | 313,349 | 0.1% | Capital | Yes |
| Rondônia | RO | 2015-2017 | 3 | 7,144 | 2,381 | 1,815,278 | 0.9% | State | No |
| Roraima | RR | 2003-2014 | 12 | 5,745 | 479 | 652,713 | 0.3% | State | Yes |
| **Northeast** |  |  |  |  |  |  |  |  |  |
| Alagoas | AL | 2010-2011 | 2 | 10,287 | 5,144 | 3,365,351 | 1.6% | State | No |
| Aracaju | SE | 2000-2016 | 17 | 43,376 | 2,552 | 672,614 | 0.3% | Capital | Yes |
| Fortaleza | CE | 2000-2015 | 16 | 93,694 | 5,856 | 2,703,391 | 1.3% | Capital | Yes |
| João Pessoa | PB | 2000-2017 | 18 | 27,578 | 1,532 | 825,796 | 0.4% | Capital | Yes |
| Natal | RN | 2000-2008 | 9 | 20,431 | 2,270 | 896,708 | 0.4% | Capital | No |
| Recife | PE | 2000-2017 | 18 | 67,708 | 3,762 | 1,661,017 | 0.8% | Capital | Yes |
| Salvador | BA | 2000-2005 | 6 | 28,424 | 4,737 | 2,900,319 | 1.4% | Capital | No |
| Teresina | PI | 2000-2006 | 7 | 6,581 | 940 | 871126 | 0.4% | Capital | No |
| **Central-West** |  |  |  |  |  |  |  |  |  |
| Campo Grande | MS | 2000-2003 \| 2008-2012 | 9 | 19,416 | 2,157 | 916,001 | 0.4% | Capital | No |
| Cuiabá | MT | 2000-2018 | 19 | 35,524 | 1,870 | 623,614 | 0.3% | Capital | Yes |
| Distrito Federal | DF | 2000-2017 | 18 | 95,658 | 5,314 | 3,094,325 | 1.5% | Federal Capital | Yes |
| Goiânia | GO | 2000-2017 | 18 | 87,693 | 4,872 | 1,555,626 | 0.7% | Capital | Yes |
| Mato Grosso (Interior) | MT | 2000-2018 | 19 | 58,249 | 3,066 | 1,923,187 | 0.9% | Interior | No |
| **Southeast** |  |  |  |  |  |  |  |  |  |
| Belo Horizonte | MG | 2000-2018 | 19 | 173,753 | 9,145 | 2,530,701 | 1.2% | Capital | Yes |
| Campinas - SMS | SP | 2010-2018 | 9 | 54,484 | 6,054 | 1,223,237 | 0.6% | Interior | No |
| Campinas - UNICAMP | SP | 2000-2005 | 6 | 9,242 | 1,540 | 1,223,237 | 0.6% | Interior | No |
| Angra dos Reis | RJ | 2007-2018 | 12 | 2,846 | 237 | 210,171 | 0.1% | Interior | No |
| Barretos (DRS) | SP | 2000-2019 | 20 | 30,947 | 1,547 | 445,216 | 0.2% | Interior | No |
| Espírito Santo | ES | 2000-2012 | 13 | 27,249 | 2,096 | 2,033,067 | 1.0% | Metropolitan region | Yes |
| Jahu | MG | 2000-2019 | 20 | 14,732 | 737 | 153,463 | 0.1% | Interior | No |
| Poços de Caldas | MG | 2007-2014 | 8 | 5,861 | 733 | 169,838 | 0.1% | Interior | No |
| Santos | SP | 2008-2011 | 4 | 10,187 | 2,547 | 433,991 | 0.2% | Interior | No |
| São Paulo | SP | 2000-2015 | 16 | 656,917 | 41,057 | 12,396,372 | 5.8% | Capital | Yes |
| **South** |  |  |  |  |  |  |  |  |  |
| Curitiba | PR | 2000-2017 | 18 | 92,399 | 5,133 | 1,963,726 | 0.9% | Capital | Yes |
| Florianópolis | SC | 2008-2016 | 9 | 27,029 | 3,003 | 516,524 | 0.2% | Capital | No |
| Porto Alegre | RS | 2000-2017 | 18 | 105,817 | 5,879 | 1,492,530 | 0.7% | Capital | Yes |
| *Available at: https://www.inca.gov.br/BasePopIncidencias/Home.action - Last updated on 25/11/2022 - Accessed on 08/02/2023 | | | | | | | | | |
| ** Population-Based Cancer Registries (PBCRs) with state or capital population coverage, with more than 10 years of historical series, starting from 2000, were selected | | | | | | | | | |
